# Optimized fistulectomy using the novel FiXcision^®^ device: a technical feasibility study and evaluation of short term healing rates

**DOI:** 10.1007/s10151-019-02025-7

**Published:** 2019-07-03

**Authors:** M. M. Kasiri, S. Riss, A. Stift, A. D. Binder, U. Kogovšek, M. Huth, I. E. Kronberger

**Affiliations:** 1 Division of General Surgery, Department of Surgery, Medical University of Vienna/AKH, Vienna, Austria; 2 Division of General Surgery, Hospital Tulln, Tulln, Austria; 3 Division of General Surgery, Department of Surgery, University of Ljubljana, Ljubljana, Slovenia; 4 Department of Visceral, Transplant and Thoracic Surgery, Center of Operative Medicine, Innsbruck Medical University, Innsbruck, Austria

## Introduction

Anal fistula influences patients’ quality of life significantly by causing pain, hygienic problems and constant perianal inflammation [1]. The main goal of surgical treatment is to close the internal fistula opening and to preserve anorectal function. Minimally invasive procedures have been developed to heal fistulas without damaging anal sphincter muscle. Coring out of the fistula tract is an essential part of some techniques. However, this may be difficult and carries the risk of injury of the sphincter muscle and incomplete removal of the tract. FiXcision^®^ is a novel instrument, designed for a controlled circumferential excision of the entire perianal fistula tract, minimizing tissue trauma to the sphincter muscle.

The aim of the present study was to assess the technical feasibly and applicability of the FiXcision^®^ device and its effect on the short-term healing rate.

## Materials and methods

Fourteen consecutive patients (5 females and 9 males, with a median age of 45 years), operated on for transsphincteric and intersphincteric fistulas of cryptoglandular origin in 2018, were included in the study.

Fistulas were classified according to the Parks classification [2]. All clinical data were obtained from local institutional databases and questionnaires and recorded prospectively. Complications were defined using the Clavien–Dindo classification [3]. Postoperative pain was evaluated with a visual analog scale (VAS) (0–10 points; 10 points indicate maximum pain). Continence status was recorded at baseline and at the first follow-up visit using the Vaizey incontinence score (VIS) [4].

This study was approved by the ethics committee of the Medical University of Vienna (vote number: 2160/2018) and conducted according to the principles of the Helsinki Declaration and Good Clinical Practice.

Exclusion criteria included a history of malignant neoplasia and/or radiation to the pelvic area, patients with immunodeficiency, Crohn’s disease, ongoing perianal sepsis with abscess and any treatment with immunomodulatory medication during the previous 4 weeks.

The primary endpoint of the study was to investigate the technical feasibility and applicability as well as safety of the FiXcision^®^ device (A.M.I. Agency for Medical Innovations, Feldkirch, Austria). In addition, the short-term healing rate of treated fistulas was assessed.

### Surgical technique

Prior to surgery any active inflammation or abscess were ruled out. Surgical procedures were performed by one experienced colorectal surgeon from each center. The operation was conducted under general anesthesia. Patients were placed in lithotomy position. All patients received single shot of Cefuroxim 1.5 g and Metronidazole 500 mg 30 min prior surgery.

**Fig. 1:**
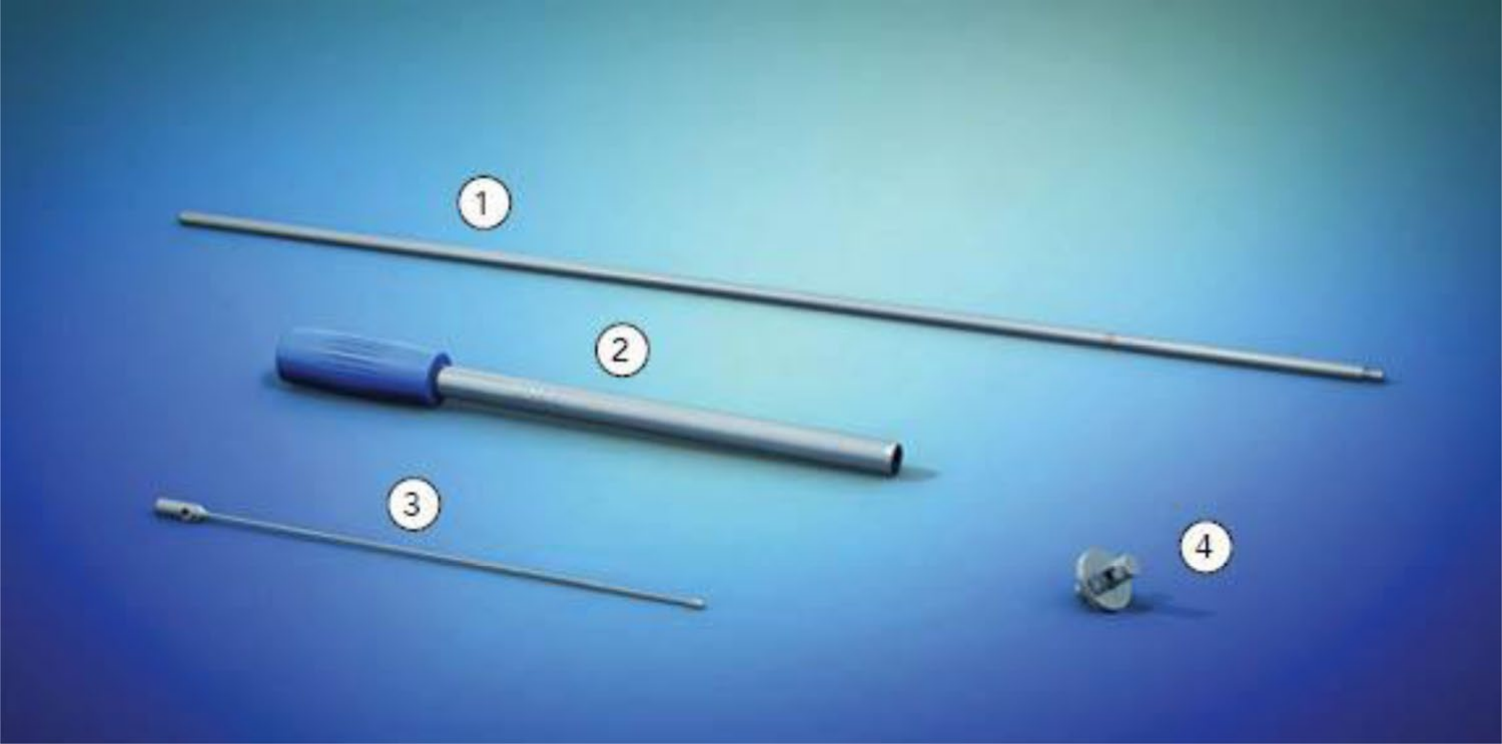
FiXcision^®^ device consisting of four parts: 1. guiding stab, 2. cylinder-shaped cutting sleeve, 3. flexible probe (guidewire), 4. stopper (picture and containing property rights belong to A.M.I. company, Austria)

**Fig. 2:**
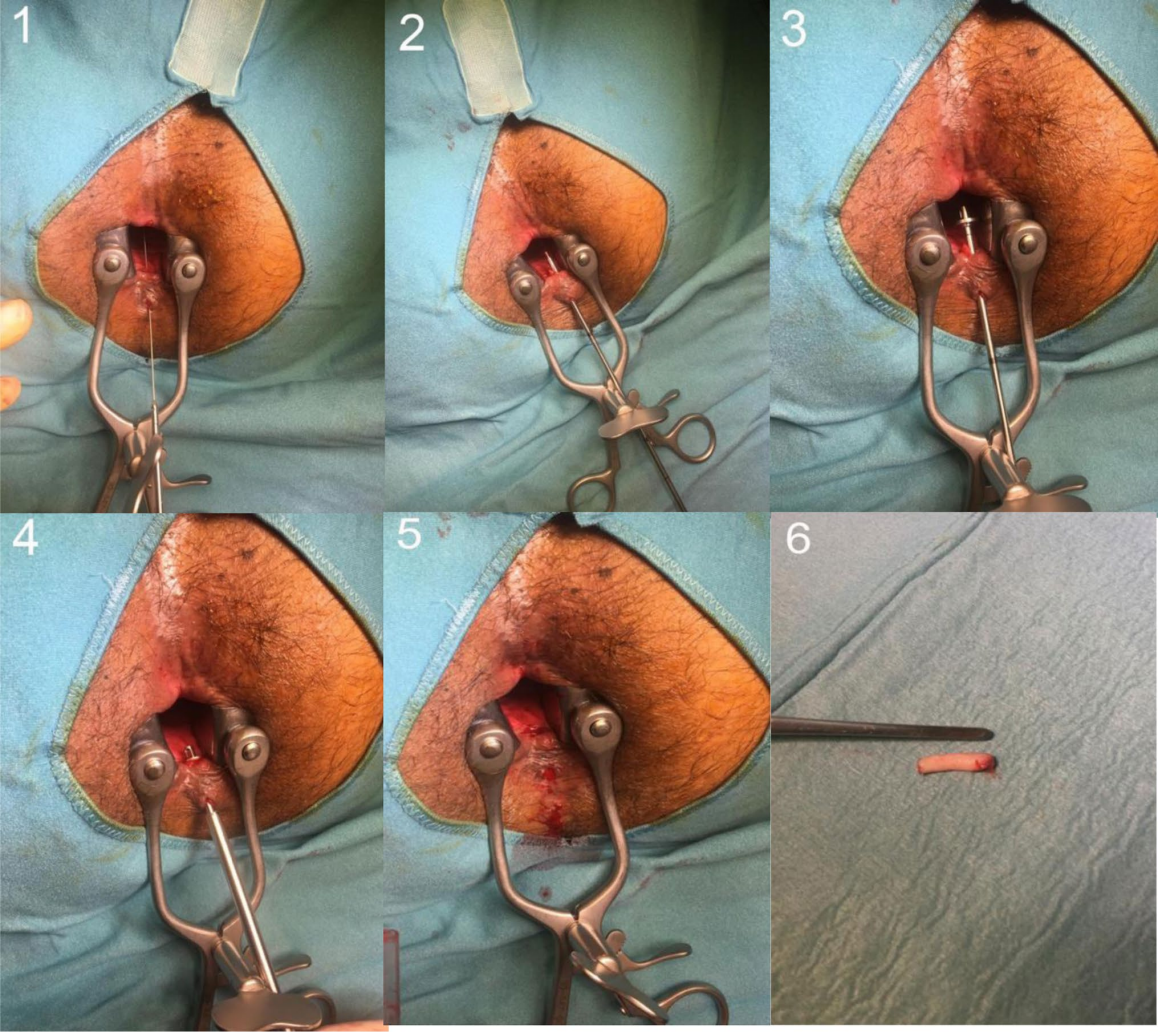
Demonstration of a fistulectomy of an anal fistula of cryptoglandular origin using the FiXcision^®^ device: 1. identification of the tract using the flexible guidewire; 2. probing the fistula tract by guiding stab and removing of the flexible probe; 3. screwing the stopper on to the guiding stab; 4. introducing the cutting sleeve along the guiding rod and pushing forward with rotary motions against the stopper; 5. pulling out the cutting sleeve together with the guiding tab after unplugging the stopper; 6. specimen of the epithelial cylinder

After anal inspection and removal of any loose setons, the FiXcision^®^ device was introduced. The device consists of four parts (Fig. 1): flexible probe (guidewire), guiding stab, cylinder-shaped cutting sleeve and a stopper. It is made of two main components: guiding rods and cutting sleeve. After inserting the guidewire into the fistula, a small circular cut around the tract is made by pushing forward the cutting sleeve with rotary motions along the guiding rods against the stopper. This enables a clean excision of the inner layer of the fistula tract after removing the cutting sleeve (Fig. 2).

After excision of the fistula tract by the FiXcision^®^ device, closure of the fistula’s internal opening is up to the surgeon’s preference. In the current series, the internal opening was either closed by an endorectal advancement flap or by primary closure using interrupted z-stitches.

## Results

Eight (57%) patients were diagnosed with transsphincteric fistulas and six (43%) with intersphincteric fistulas. The median duration of the fistula was 5 (range 2–8 months) months.

In two (14%) patients, the transsphincteric fistula was excised incompletely by FiXcision^®^ and a fistulectomy with primary sphincter repair was performed. In all other patients, a complete fistula tract specimen without macroscopically obvious sphincter muscle was achieved. Moreover, no surgical complications were observed during or after the operation. Endorectal advancement flaps and z-stitches were used to close the internal opening in nine (64%) and five (35%) patients, respectively. The median operation time was 20 min (range 6–45 min). Median hospital stay was 1 day (range 1–4 days). Intraoperative details are outlined in Table 1.

**Table 1:** Surgical details of included patients

Patient number	Type of fistula	Fistula (*n*)	Length of fistula tract (cm)	Internal opening (*n*)	External opening (*n*)	Operation time (min)	Intraoperative complications	Hospital stay (days)	Additive technique
Intraoperative details									
1	Intersphincteric	1	4	1	1	15	0	1	Z-stitch
2	Transsphincteric	1	2	1	1	19	0	1	Z-stitch
3	Intersphincteric	1	2	1	1	7	0	1	Z-stitch
4	Intersphincteric	1	1.3	1	1	23	0	4	Z-stitch
5	Intersphincteric	1	2	1	1	20	0	4	Mucosa flap
6	Intersphincteric	2	2	1	1	45	0	2	Mucosa flap
7	Transsphincteric	1	1.8	1	1	6	0	1	Mucosa flap
8	Transsphincteric	1	3	1	1	30	0	1	Mucosa flap
9	Transsphincteric	1	2.5	1	1	30	0	2	Mucosa flap
10	Intersphincteric	1	2	1	1	45	0	1	Mucosa flap
11	Transsphincteric	1	5	1	1	20	0	1	Mucosa flap
12	Transsphincteric	1	3.6	1	1	16	0	1	Z-stitch
13	Transsphincteric	1	2	1	1	20	0	1	Mucosa flap
14	Transsphincteric	1	2	1	1	15	0	1	Mucosa flap

Complete healing implying no clinical signs of discharge or abscess formation was observed in eight patients (66%) and incomplete healing in four patients (34%). The healing rates of transsphincteric and intersphincteric fistulas were comparable. The two patients who had a primary unsuccessful fistulectomy using the new device were excluded from follow-up.

The median VAS score for postoperative pain was 0 (range 0–1).

None of the patients experienced symptoms of fecal incontinence postoperatively with a median Vaizey incontinence score of 0 (range 0–2).

## Discussion

FiXcision^®^ is one of the latest surgical innovations, developed for treating anorectal fistula. With the device, it is possible to perform a complete circumferential cut around the granulation and epithelial tissue along the entire tract in a controlled manner. FiXcision^®^ was designed to reduce the incision diameter without harming surrounding sphincter muscle. Najarian et al. reported a similar idea in 2010 [5]. They described a minimally invasive approach for core-out excision of the fistula tract using a rigid fistulectomy set in one patient suffering from two anal fistulas. The device consisted of six rigid parts and was designed to reduce the diameter of the fistulectomy lumen to minimize damage to the anal sphincter.

FiXcision^®^ was developed with the goal minimizing the risk of intraoperative sphincter damage while performing a safe fistulectomy and to improve the outcome of standardized surgical techniques. Our study is the first to evaluate the applicability and short-term efficacy of this new device for treating anorectal fistula.

From the technical point of view the device is easy to use, especially for straight fistulas. No technical difficulties or complications using the device were reported by surgeons at the four hospitals in the study. Notably, longer and curved fistula tracts may be more challenging to core out as the device is rigid. However, in the majority of patients in the present series, a complete fistula tract specimen could be obtained. Only two patients had particularly large transsphincteric fistulas, which resulted in incomplete specimens.

The device offers another potential benefit. In addition to a clear and precise cut around the tract without involving any sphincter muscle, it enables a careful exploration of the entire tract, which could lead to the detection of additional second tracts. Those tracts are usually easily missed during simple fistulectomy.

The initial clinical results and healing rates of fistulectomy using the FiXcision^®^ device are encouraging. We observed no significant postoperative pain shortly after surgery or at the follow-up visit at 1 month. Furthermore, no perioperative complications were observed and the included patients did not report any symptoms of minor or major fecal incontinence.

It is noteworthy that FiXcision^®^ represents only one part of the fistula treatment strategy, as the internal opening requires additional closure by other techniques. It is also important to mention that using the device, the internal opening might become slightly bigger in those patients with only small openings. However, none of the participating surgeons mentioned any significant problems in closing internal openings.

A better overall healing rate is another major goal of new fistula treatment devices. In the present study, the primary aim was to show the feasibility and safety of the device. Taking into account that the choice of the technique used to close the tract depended on the surgeon’s preference, there was a 66% complete healing and 34% incomplete healing rate. Further studies with a large number of homogeneous patients will be necessary to address the question whether the FiXcision^®^ device improves fistula healing rates.

To prove the applicability of the new device, we included intersphincteric anal fistulas, which by definition can be treated by simple fistulotomy alone. FiXcision^®^ may have a role in this group of patients too, as it avoids any sphincter damage.

## Conclusions

The FiXcision^®^ technique is simple and safe and may also facilitate detection of secondary fistula tracts and improve fistula healing rates. Further studies will be required to clarify the role of this new device.
